# Spatiotemporal accumulation of fatal pharyngeal entrapment of flatfish in harbour porpoises (*Phocoena phocoena*) in the German North Sea

**DOI:** 10.7717/peerj.10160

**Published:** 2020-10-20

**Authors:** Stephanie Gross, Marco Roller, Holger Haslob, Miguel Grilo, Jan Lakemeyer, Anja Reckendorf, Peter Wohlsein, Ursula Siebert

**Affiliations:** 1 Institute for Terrestrial and Aquatic Wildlife Research (ITAW), University of Veterinary Medicine Hannover, Foundation, Büsum, Germany; 2 Thünen-Institute of Sea Fisheries, Bremerhaven, Germany; 3 CIISA—Centre for Interdisciplinary Research in Animal Health, University of Lisbon, Lisbon, Portugal; 4 Department of Pathology, University of Veterinary Medicine Hannover, Foundation, Hanover, Germany

**Keywords:** Odontocetes, Common sole, Asphyxiation, Bolus death, Post-mortem examination

## Abstract

The evolution of a permanent separation of the upper respiratory and digestive tract is one of the adaptions cetaceans evolved for their aquatic life. Generally, it prevents odontocetes from choking on either saltwater or foreign bodies during ingestion under water. Nevertheless, several sporadic single case reports from different parts of the world show that this separation can be reversed especially by overly large items of prey. This incident can have a fatal outcome for the odontocetes. The German federal state of Schleswig-Holstein has a year-round, permanent and systematic stranding network that retrieves stranded marine mammals from its shorelines and constantly enables post-mortem examinations. In 2016, with nine affected animals, a high incidence of fatal pharyngeal entrapment of flatfish in harbour porpoises (*Phocoena phocoena*) occurred during spring and early summer on the German North Sea island of Sylt. All flatfish were identified as common sole (*Solea solea*). A retrospective post-mortem data analysis over a 30-year period from the North and Baltic Sea revealed similar yearly and seasonally case accumulations on the same island in the 1990s as well as several single case events over the whole timespan. All cases except one were caused by flatfish. When flatfish speciation was performed, only common sole was identified. From 1990 to 2019, of all examined harbour porpoises, 0.3% (2/713) from the Baltic Sea and 5.5% (45/820) from the North Sea died due to fish entrapped in the pharynx. On the North Sea coast, the occurrence of fatal obstruction shows high yearly variations from 0 to 33.3%. Years that stand out are especially 1990 to 1992, 1995, as well as 2016. The majority of all cases generally occurred between April and July, indicating also a seasonality of cases. This study evaluates the occurrence of fatal pharyngeal entrapment of fish in two geographically separated harbour porpoise populations. Additionally, common sole is clearly identified as a potentially risky item of prey for these small odontocetes.

## Introduction

Marine mammals evolved several anatomical adaptations due to their aquatic life. In odontocetes, one specific adaptation is the complete separation of the airways and the digestive tract (Reidenberg & Laitman, 1987). An elongated larynx traverses the digestive tract at the level of the pharynx. The larynx is permanently positioned by a palatopharyngeal sphincter muscle in the internal nares which lead up to the blow hole on top of the head. This separation of the upper respiratory and digestive tracts allows odontocetes to feed under water without a restriction in echolocation or the risk of choking water or foreign bodies. However, this causes an anatomical bottleneck for the prey and additionally restricts the size of items of prey as these must pass the larynx on one or the other side (Reidenberg & Laitman, 1987), although an asymmetry in the position of the larynx creates a larger food channel on its right side (Macleod et al., 2007). Another potential risk is the fact that most odontocetes do not masticate their prey but swallow it whole and often alive (Werth, 2000). For this reasons, either agile prey which tries to escape or overly large prey may cause a dislocation of the larynx. If this happens, an obstruction and impeded re-articulation of the larynx makes breathing impossible for the cetacean, the consequence being lethal hypoxia and suffocation.

There are several single case reports of different odontocetes with fatal entrapment of fish coming from various parts of the world including one beluga whale (*Delphinapterus leucas*) in Alaska (Rouse, Burek-Huntington & Shelden, 2018), two long-finned pilot whales (*Globicephala melas*) in the Netherlands (IJsseldijk et al., 2015), two Indio-Pacific bottlenose dolphins (*Tursiops aduncus*) in Australia (Byard et al., 2003; Byard et al., 2010), two common bottlenose dolphins (*Tursiops truncatus*) in the USA (Watson & Gee, 2005; Mazzoil et al., 2008) and one in Puerto Rico (Mignucci-Giannoni et al., 2009), two Guiana dolphins (*Sotalia guianensis*) in Brazil (Domiciano et al., 2016; Mariani et al., 2020) as well as two harbour porpoises in the USA (Scheffer & Slipp, 1948; Scheffer, 1953) and one in Belgium (Haelters, Kerckhof & Jauniaux, 2018). So far, only one study conducted a retrospective evaluation over a 15-year period in two populations of common bottlenose dolphins of the Indian River Lagoon, Florida, USA (Stolen et al., 2013) showing fatal entrapment of fish as the cause of death in 4% of the carcasses in one population, while no cases where observed in the other population. In many of these reports, the prey item was reported to be too large to swallow (Byard et al., 2003; Watson & Gee, 2005; Mignucci-Giannoni et al., 2009; Domiciano et al., 2016; Rouse, Burek-Huntington & Shelden, 2018; Mariani et al., 2020). Other reports suggested that the spines of the prey species or unusual prey species accounted for the fatal obstruction (Mignucci-Giannoni et al., 2009; Stolen et al., 2013; IJsseldijk et al., 2015; Haelters, Kerckhof & Jauniaux, 2018). Flatfish occurred in one report as causative agent (Rouse, Burek-Huntington & Shelden, 2018) and specifically common sole was named on one other occasion (IJsseldijk et al., 2015).

The present study describes a spatiotemporal accumulation of fatal pharyngeal entrapment of common sole in nine harbour porpoises. The small cetaceans were all found stranded dead on the German North Sea island of Sylt between April and the beginning of July 2016. Furthermore, a retrospective analysis of necropsy data over a 30-year period in Schleswig-Holstein revealed similar strandings in the same area of the North Sea in the years 1990 to 1992, and 1995. In contrast, only two cases occurred in the harbour porpoise population of the German western Baltic Sea over the same period. This study evaluates spatiotemporal changes in cases of fatal pharyngeal entrapment of fish in harbour porpoises of the German North Sea. Furthermore, it compares the occurrence of cases in two geographically separated harbour porpoise populations over a 30-year time period.

## Material and Methods

Necropsies of marine mammals are routinely performed at the Institute for Terrestrial and Aquatic Wildlife Research (ITAW), University of Veterinary Medicine Hannover, Foundation, Büsum, Germany, as part of the stranding network of the German federal state of Schleswig-Holstein (Benke et al., 1998; Siebert et al., 2006). In 2016, 86 post-mortem examinations of harbour porpoises from the North and Baltic Sea coast were performed. Of these, 11 cases showed an obstruction of the upper respiratory tract in combination with a dislocated or compressed larynx due to ingested fish. Of the 11 animals, nine derived from the North Sea, one from the Baltic Sea and for one animal, the stranding location was not noted. In the following, we focus on the nine North Sea carcasses (cases no. 1–9) that were all retrieved from the island of Sylt within a three-month period. These nine animals were stored frozen prior to necropsy. Their decomposition states varied from fresh (*n* = 1), to good (*n* = 2), moderate (*n* = 3) and advanced decomposition (*n* = 3).

Pathological investigations were generally performed in accordance with a standardised protocol (Siebert et al., 2001; IJsseldijk, Brownlow & Mazzariol, 2019) but were partly or widely restricted for animals in moderate and advanced decomposition. A full post-mortem examination was performed in four carcasses (cases 1, 2, 7, 9), while it was restricted in two (cases 6, 8) and unfeasible in three carcasses (cases 3, 4, 5) due to their advanced states of decomposition. In the latter three cases, if feasible, the examination included gender and total length determination, as well as pharynx inspection for foreign bodies and stomach content collection. For age determination, six teeth from the lower jaw were removed and annual growth layers were counted following established protocols (MyrickJr et al., 1983; Lockyer, 1995). When age determination via the teeth could not be performed, animals were classified into the following age classes depending on their total length: juvenile (1.0–1.3 m) and adult (>1.3 m). The nutritional status of the carcasses was judged depending on the blubber thickness and muscle development (Siebert et al., 2001). For the three carcasses in an advanced stage of decomposition, the nutritional status could not be determined due to fat and tissue autolysis. Depending on the decomposition status, histopathological samples were taken from organs and tissues with morphological changes. Histopathological samples were fixed in 10% buffered formalin, embedded in a paraffin wax, sectioned at 3 µm and stained with haematoxylin and eosin. The flatfish were measured to determine their length, width, height and weight. Fish species were identified by morphological parameters based on comparison to an identification guide (Muus & Nielsen, 2013).

Retrospective data analysis was performed using the post-mortem database of the ITAW which included 1,533 post-mortem records of harbour porpoises from the North and Baltic Sea coasts of Schleswig-Holstein from 1990 to 2019. This analysis included carcasses that had undergone a complete necropsy as well as carcasses that had only been partially investigated due to their advanced state of decomposition. In all included cases (the ones with and without fish entrapped in the pharynx), at least the upper respiratory tract had been assessed.

**Figure 1:**
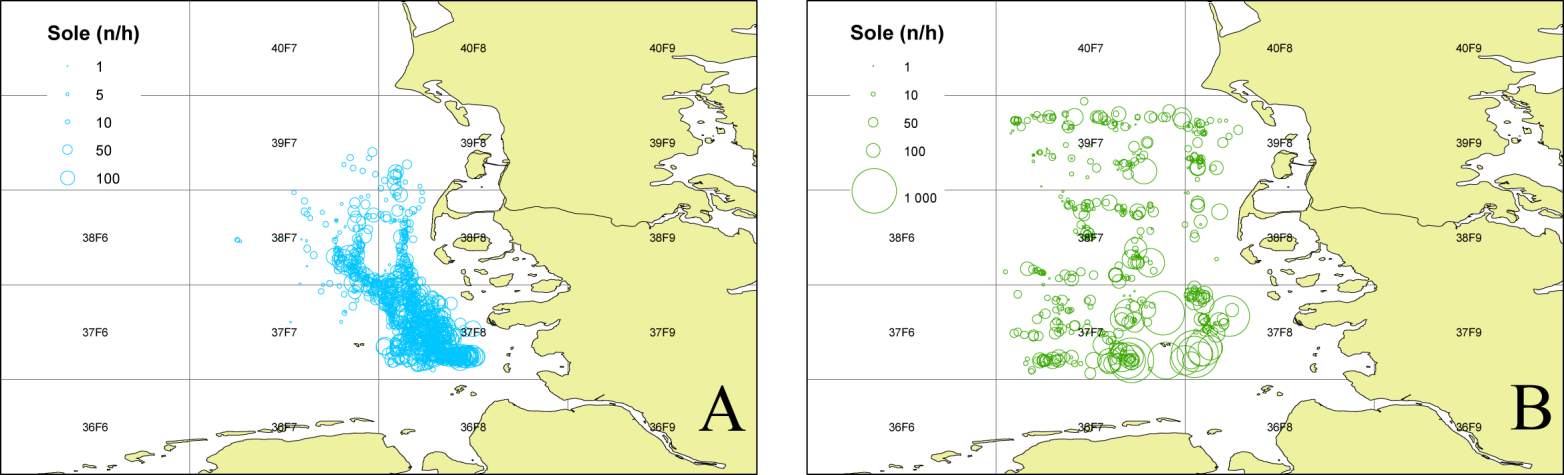
Sole abundance in the German Bight. Sole number per trawling hour (n/h) for the German sole survey (1976–2012 2nd quarter (A)) and the North Sea Beam Trawl Survey (1985–2019 3rd quarter; (B)) for each station over the whole time series. The grid displays the ICES statistical rectangles in the investigation area.

To determine the occurrence of common sole two sole abundance indices (mean n per hour trawling) were calculated. The 2nd quarter index (April–June) is based on the German sole survey (Damm & Weber, 1990; ICES, 2015) which was conducted during sole spawning time in the coastal area of the German Bight (1976–2012). For this index only stations with maximum water depths of 25m were used, to account for consistency over the whole time period. The 3rd quarter index (August–September) is based on data from the North Sea beam trawl survey (ICES, 2020a; ICES, 2020b) which operates farther offshore. For both indices only data from the ICES statistical rectangles 39F7, 39F8, 38F7, 38F8, 37F7, 37F8 (Fig. 1) were utilized to account for the investigation area in the German Bight of the present study. The ICES statistical rectangle coding system provides a spatial grid covering the North-East Atlantic and its adjacent seas. The latitudinal rows have intervals of 30′ and the longitudinal columns have intervals of 1° (see Fig. 1 (ICES, 1977)).

## Results

**Figure 2:**
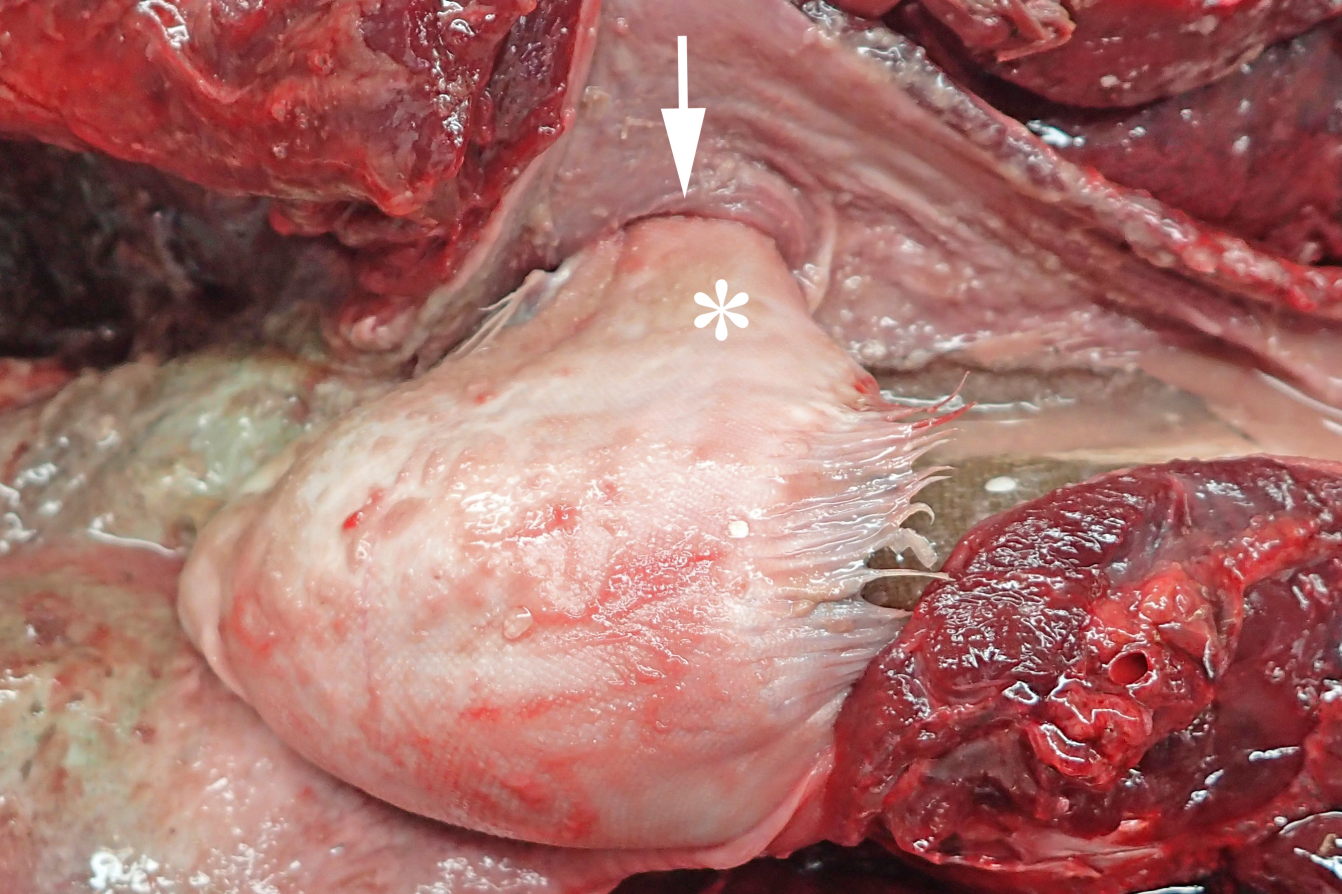
Common sole stuck headfirst in the internal nares of a harbour porpoise (case 2). The arrow indicates the entrance to the internal nares, the asterisk marks the underside of the common sole. Photo credit: Institute for Terrestrial and Aquatic Wildlife Research.

In 2016, a total of 37 harbour porpoises from the North Sea coast of Schleswig-Holstein were examined at the ITAW. Fatal pharyngeal entrapment of common sole was diagnosed as the cause of death in 24.3% (9/37) of these carcasses. All nine animals had been stranded on the Island of Sylt. All of these strandings took place within three months, from 7 April to 7 July, with a peak in May (7/9). The affected animals included five females and four males. Two of the females were subadults, all other animals were adults. In all of these harbour porpoises, post-mortem examination revealed a flatfish in the pharynx. In seven of the nine cases (cases 1–3, 5–8) the fish partly stuck in the nasal passage, completely obstructing the lumen and irreversibly dislodging the larynx from its normal anatomical position (Fig. 2). In six of these seven cases (cases 1–3, 6–8), the head of the fish was located in the internal nares. In one of these cases (case 7), the fish tail additionally stuck in the larynx. In the seventh case (case 5), it was not documented which end of the fish was in the internal nares. Of the remaining two cases, one (case 4) had the fish head first inside the trachea down to the bifurcation. In the last case (case 9), the head and tail of a flatfish were rostral to the larynx, while the bent fish body stuck in the caudal part of the oral cavity of the harbour porpoise. In one harbour porpoise (case 2), two additional flatfish were found in the oesophagus, one being located at the same level as the fish that stuck in the internal nares, the other one caudal to the obstruction. The latter fish was the only one showing evidence of digestion. Stranding data on the harbour porpoises are summarised in Table 1.

**Table 1:** Data of the nine harbour porpoises with fatal pharyngeal entrapment retrieved from the German North Sea island of Sylt in 2016. Stranding date, sex, age, nutritional and decomposition status as well as location of the flatfish in the upper digestive/respiratory tract.

**Case**	**Strandingdate**	**Sex**	**Age (years)**	**Nutritional status**	**Decomposition status**	**Localisation of the flatfish**
1	16∕04∕07	female^a^	7–8	good	fresh	flatfish in the pharynx, head in the internal nares
2	16∕05∕05	male	9–10	moderate	good	flatfish in the pharynx, head in the internal nares; two more are located in the cranial oesophagus
3	16∕05∕05	female	3	–	advanced	flatfish in the pharynx, head in the internal nares
4	16∕05∕15	male	11–12	–	advanced	flatfish in the trachea down to the bifurcation
5	16∕05∕16	female^a^	5–6	–	advanced	flatfish in the pharynx, partly in the internal nares
6	16∕05∕17	male	5–6	moderate	moderate	flatfish in the pharynx, head in the internal nares
7	16∕05∕18	male	6–7	good	moderate	flatfish in the pharynx, head in the internal nares and tail in the larynx
8	16∕05∕21	female	3	good	moderate	flatfish in the pharynx , head in the internal nares
9	16∕07∕07	female	8	moderate	good	flatfish body is flapped in the mouth, head and tail a few centimetres cranial to the larynx

**Notes.**

a pregnant.

**Figure 3:**
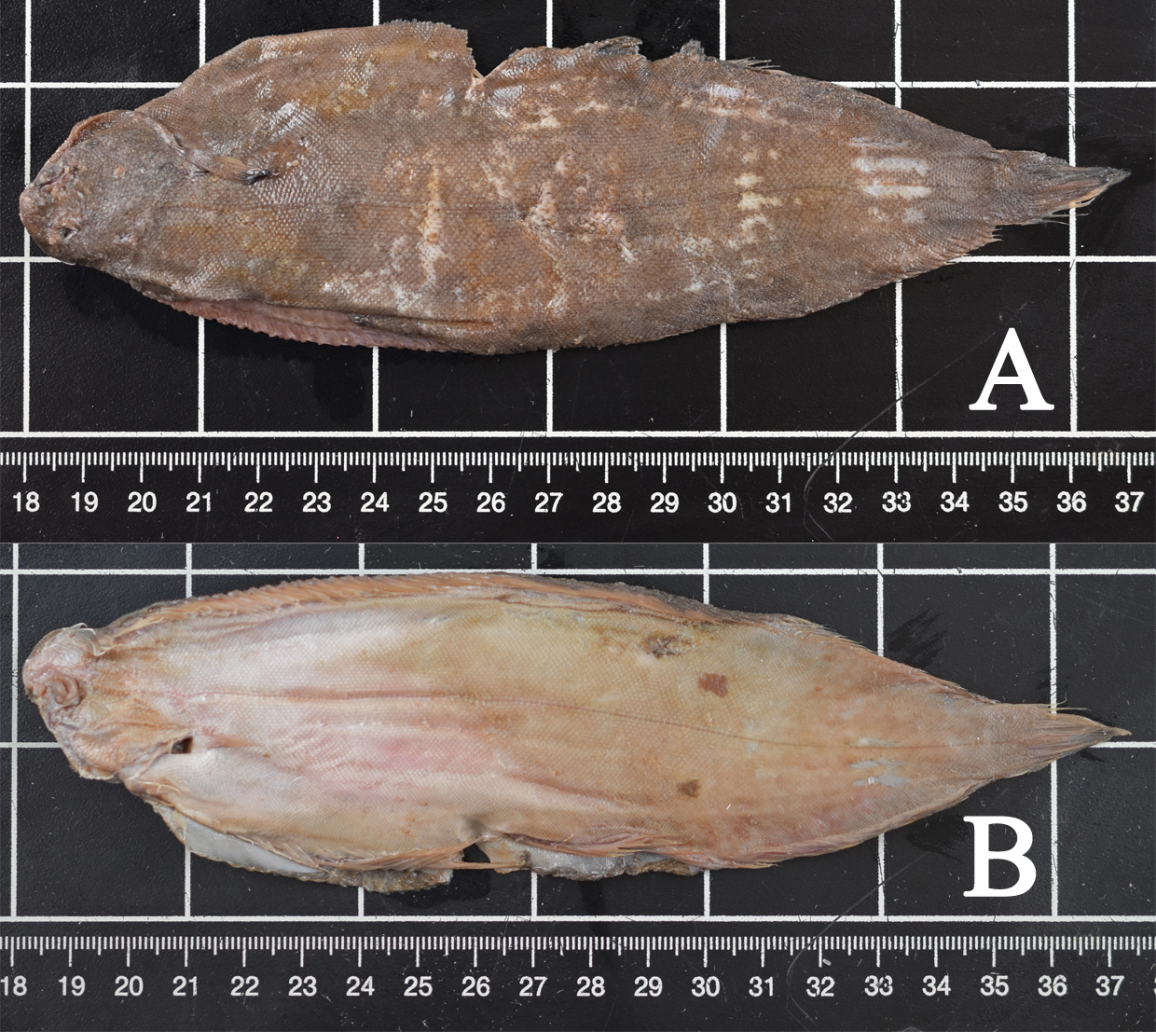
Common sole (case 7). Typical characteristics include right-sided eyes, round muzzle, jutting upper jaw, small filiform, sensory warts around the mouth, blackish pectoral fin on the upper side and pectoral fins of both sides of similar length. (A) Upside, (B) underside. Photo credit: Institute for Terrestrial and Aquatic Wildlife Research.

**Figure 4:**
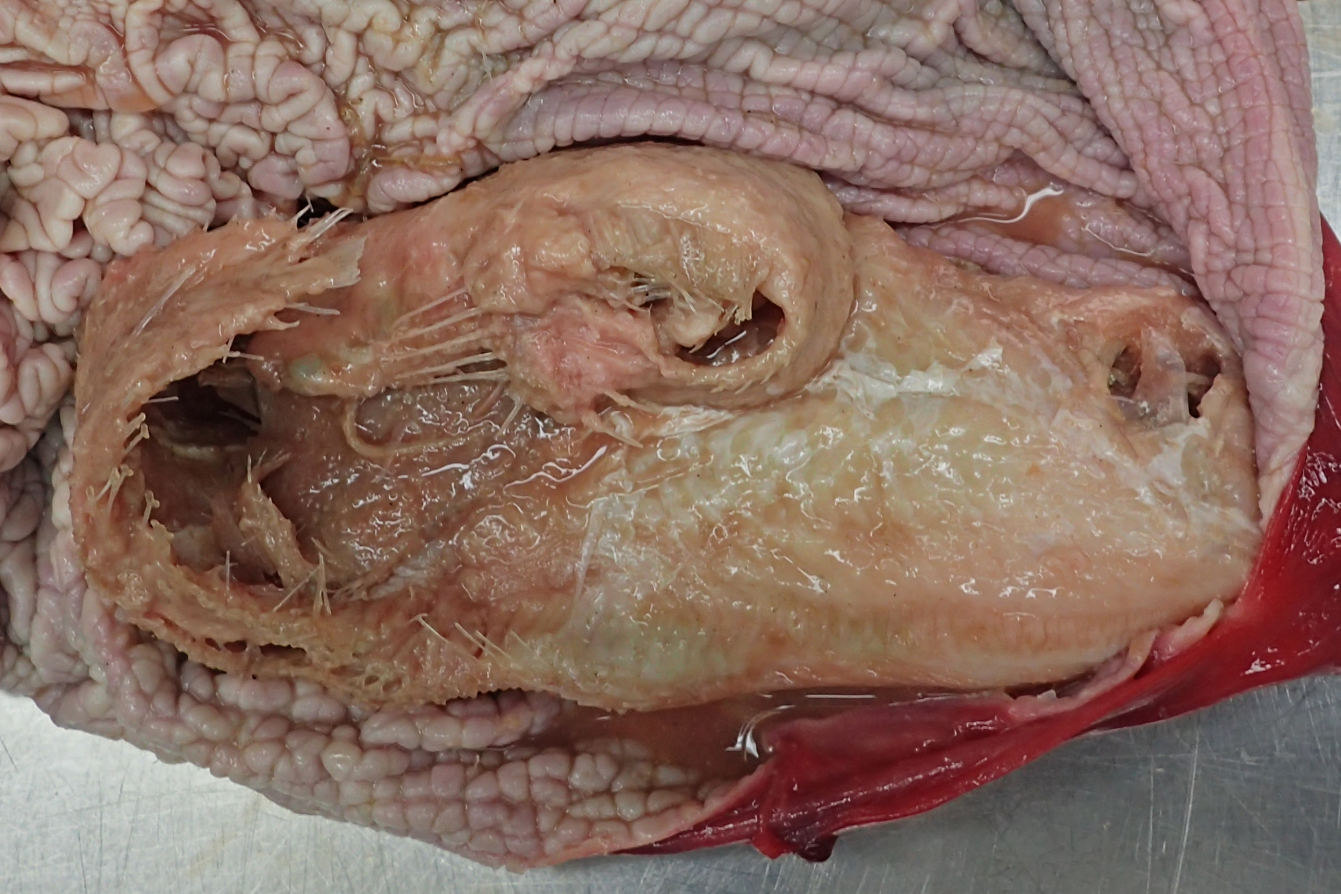
Two partly digested flatfish in the forestomach of case 1. Photo credit: Institute for Terrestrial and Aquatic Wildlife Research.

All fish were macroscopically identified as common sole (*Solea solea*) (Fig. 3). All fish causing the obstruction were in good preservation status showing no evidence of digestion following potential regurgitation. The soles varied in total length from 18.3 to 28.8 cm and in width from 5.0 to 10.7 cm. Two fish (cases 3 and 4) were missing the tail fin and therefore their total length could not be measured. The fish weighed between 28.97 and 173.25 g. Morphological data of all examined fish are shown in Table 2. In seven cases (cases 1, 2, 4–7, 9), the stomachs of the harbour porpoises were filled with different amounts of food, also including only partially digested flatfish (Fig. 4).

**Table 2:** Morphometric data of the flatfish found in the pharynges of the nine harbour porpoises from the German North Sea island of Sylt in 2016.

**Case**	**Fish species**	**Length (cm)**	**Width (cm)**	**Hight (cm)**	**Weight (g)**	**Comments**
1	*Solea solea*	21.0	6.4	0.8	67.26	–
2	*Solea solea*	25.9	9.5	1.4	122.97	fish partly in nasal passage
2	*Solea solea*	24.4	7.5	1.3	100.80	fish next to larynx
2	*Solea solea*	18.3	6.2	0.7	51.71	fish caudal to obstruction, partly digested
3	*Solea solea*	>15.4	5.0	0.9	35.37	tail fin partly missing
4	*Solea solea*	>15.2	5.0	1.0	36.65	tail fin partly missing
5	*Solea solea*	19.5	5.1	0.9	39.70	-
6	*Solea solea*	19.7	5.8	1.4	49.73	-
7	*Solea solea*	18.8	5.3	0.9	37.24	-
8	*Solea solea*	16.7	5.0	0.9	28.97	-
9	*Solea solea*	28.8	10.7	1.5	173.25	–

Histopathology was performed in five animals (cases 1, 2, 7, 8, 9) but was partially restricted due to advanced decomposition. Main histopathological findings included gastritis in four (cases 2, 7, 8, 9) and pneumonia in three animals (cases 2, 7, 9). The gastritis was in most cases of a granulomatous and an eosinophilic character and associated with endoparasitosis. One case of gastritis (case 9) was graded as severe, the other three as moderate. Severe, chronic, granulomatous and eosinophilic pneumonia associated with parasitic infestation was evident in three animals. Additional findings included multifocal fibrosis and calcification in the lung, alveolar and interstitial pulmonary oedema, as well as pulmonary emphysema. One animal had multifocal crater-shaped erosions in the oesophageal tissue without inflammatory reaction. Another one displayed moderate, chronic cholangitis and pancreatic duct inflammation. Most of these findings were related to parasitic infestations of the corresponding organs with the lung and stomach being most frequently affected. In two animals the cerebrum was examined but did not have pathological changes. In none of the carcasses were the additional pathological findings identified as the cause of death. Histopathological findings are summarised in Table 3.

**Table 3:** Summary of histopathological findings in six of the nine harbour porpoises with fatal pharyngeal entrapment of common sole from the German North Sea island of Sylt in 2016. Due to advanced decomposition, the examination of cases 3–5 was limited to gender and total length determination, as well as pharynx inspection for foreign bodies and stomach content collection. Non-investigated organs are marked with n.a. (not assessed).

**Case**	**1**	**2**	**3**	**4**	**5**	**6**	**7**	**8**	**9**
**Macroscopic organ inspection**	yes	yes	no	no	no	yes	yes	yes	yes
**Histopathology**	yes	yes	no	no	no	no	yes	yes	yes
**Missing organs**	no	no	no	no	no	yes ^∗1^	yes ^∗2^	no	no
**Pneumonia**	no	+++	n.a.	n.a.	n.a.	n.a.	+++	no	+++
**Lung fibrosis**	+	no	n.a.	n.a.	n.a.	n.a.	no	++	no
**Lung calcification**	no	yes ^∗3^	n.a.	n.a.	n.a.	n.a.	no	++	yes ^∗3^
**Alveolar/interstitial oedema**	+	+++	n.a.	n.a.	n.a.	n.a.	++	+++	+++
**Lung emphysema**	+	no	n.a.	n.a.	n.a.	n.a.	no	no	no
**Oesophageal erosions**	no	no	n.a.	n.a.	n.a.	n.a.	yes ^∗3^	no	no
**Gastritis**	no	++	n.a.	n.a.	n.a.	n.a.	++	++	+++
**Cholangitis**	++	no	n.a.	n.a.	n.a.	n.a.	no	no	no
**Pancreatic duct inflammation**	++	no	n.a.	n.a.	n.a.	n.a.	no	no	no
**Lung parasites**	+	+	n.a.	n.a.	n.a.	++	++	+++	+++
**Heart parasites**	no	no	n.a.	n.a.	n.a.	no	no	n.a.	+
**Stomach parasites**	++	+++	n.a.	n.a.	n.a.	no	++	+	+
**Liver parasites**	++	no	n.a.	n.a.	n.a.	n.a.	no	no	no
**Pancreas parasites**	+	no	n.a.	n.a.	n.a.	n.a.	no	no	no
**Ear parasites**	++	yes ^∗3^	n.a.	n.a.	n.a.	n.a.	++	n.a.	+++

**Notes.**

+ mild.

++ moderate.

+++ severe.

*1 right lung, all abdominal organs except stomach.

*2 both eyes.

*3 severity not specified.

**Figure 5:**
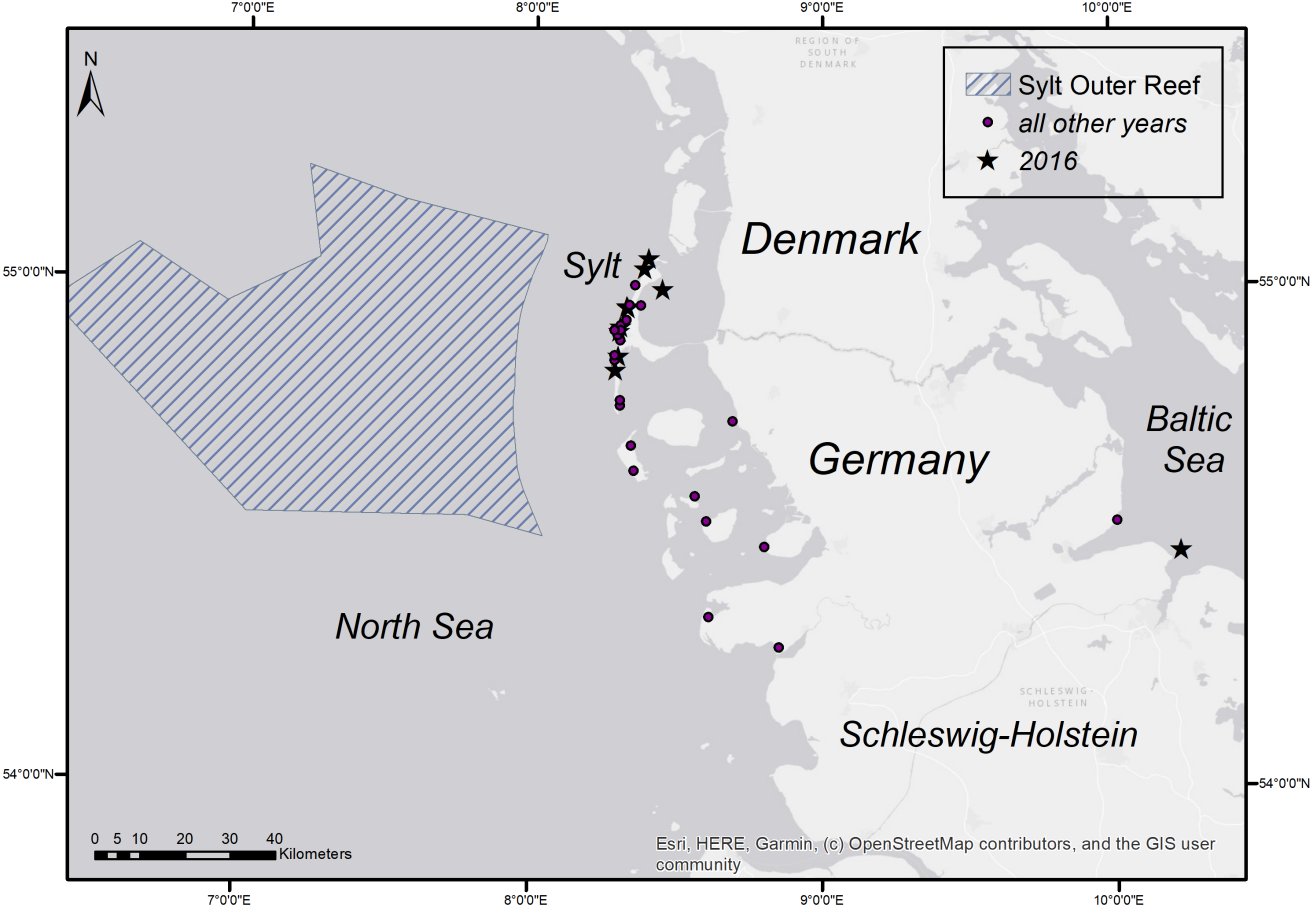
Stranding locations of harbour porpoises with fatal pharyngeal entrapment of fish derived from the coasts of Schleswig-Holstein from 1990 to 2019. The map was created using ArcGIS for Desktop 10.5 (©Esri, Inc., Redlands, CA, USA).

A retrospective evaluation from 1990–2019 included a total number of 1,533 necropsied harbour porpoises from the Schleswig-Holstein’s North and Baltic Sea costs of which 48 individuals (3.4%) showed fatal pharyngeal entrapment of fish (Table 4). The majority of these animals (*n* = 36, including the nine animals from 2016) were again retrieved from Sylt. Nine animals came from other areas at the North Sea coast, one’s origin was unknown and only two were from the Baltic Sea coast. Focusing on the North Sea coast, there are four years, 1990–1992 and 1995 that showed a higher occurrence of cases in addition to 2016. Of all harbour porpoises examined from the North Sea coast, a total of 33.3% (2/6) in 1990, 28.6% (6/21) in 1991, 26.3% (5/19) in 1992, and 28.6% (6/21) in 1995 had died due to pharyngeal entrapment of fish. For the other years, the percentage was between 0 and 11.1 (Table 5). From 1990 to 2019, in total, 5.5% (45/820) of all harbour porpoises examined from the North Sea coast had died due to this phenomenon. The occurrence in the Baltic Sea over the same period was only 0.3% (2/713). The stranding locations of cases with pharyngeal entrapment of fish are shown in Fig. 5. All fish found in the pharynx were flatfish except one case from the Baltic Sea which was identified as cod (*Gadus morhua*). In the latter one, the large size of the fish had most probably caused the obstruction. Common sole was morphologically identified in 23 of the total 47 flatfish cases (45 from the North Sea, one from the Baltic Sea, one of unknown origin). Of the affected harbour porpoises, 30 (62.5%) were female and 18 (37.5%) male. Determined ages varied between two and 17 years. Of the 30 females, 25 were adults (≥4 years) of which nine were pregnant and two were lactating. The majority of the cases (82.9%) occurred between April and July, with May having the highest incidence (34.1%) (Table 6). In addition to the two investigated cerebrums from 2016, six other brains of harbour porpoise with fatal pharyngeal entrapment of fish were histologically examined (in four cases including the brain stem) but no pathological changes were found.

**Table 4:** Database data for harbour porpoises with fatal pharyngeal entrapment of fish from 1990 to 2019 (excluding the nine cases from the island of Sylt in 2016).

**No.**	**Stranding date**	**Stranding location**	**Sex**	**Nutritional status**	**Decomposition status**	**Age (years)/age class**	**Fish family or species**	**Localisation of the fish**
1	90/–/–	North Sea, Sylt	male	–	severe	4.00	Common sole	pharynx
2	90∕07∕01	North Sea, North Friesland	male	–	severe	3.25	Common sole	–
3	91∕05∕31	North Sea, Sylt	female^*^	moderate	good	4.00	Common sole	pharynx/nasal passage
4	91∕06∕01	North Sea, Sylt	male	–	moderate	5.75	Common sole	pharynx
5	91∕06∕07	North Sea, Amrum	female	good	severe	4.00	Common sole	pharynx
6	91/–/–	North Sea, Sylt	male	–	severe	2.25	flatfish	–
7	91∕08∕01	North Sea, Sylt	male	moderate	moderate	5.00	flatfish	pharynx
8	91/–/–	North Sea, North Friesland	male	–	moderate	4.30	Common sole	pharynx
9	92∕04∕17	North Sea, Sylt	female	good	moderate	3.75	flatfish	–
10	92∕07∕18	North Sea, North Friesland	female	–	moderate	adult	Common sole	pharynx/nasal passage
11	92∕08∕17	North Sea, Sylt	male	–	severe	adult	flatfish	pharynx
12	92∕09∕16	North Sea, Sylt	female	–	moderate	adult	Common sole	pharynx/nasal passage
13	92∕09∕17	North Sea, St. Peter Ording	male	–	severe	adult	Common sole	pharynx/nasal passage
14	93∕05∕04	North Sea, Sylt	female	n.a.	good	adult	flatfish	pharynx/nasal passage
15	93∕06∕07	North Sea, Sylt	male	–	moderate	2.00	Common sole	pharynx
16	93/–/–	North Sea, Sylt	female	n.a.	severe	6.50	flatfish	pharynx/nasal passage
17	95∕04∕03	North Sea, Sylt	female^*^	n.a.	severe	9.50	flatfish	–
18	94∕05∕01	North Sea, Amrum	female	good	moderate	3.00	flatfish	–
19	94∕06∕01	North Sea, Sylt	female	good	good	9.00	flatfish	–
20	94∕09∕27	North Sea, Sylt	female^**^	good	moderate	3.50	flatfish	–
21	95∕05∕08	North Sea, Sylt	female^*^	good	moderate	10.00	Common sole	pharynx
22	95∕06∕08	North Sea, Sylt	female	n.a.	severe	13.00	flatfish	–
23	95/–/–	North Sea, Sylt	female^**^	n.a.	severe	17.00	flatfish	pharynx
24	95∕07∕05	North Sea, Sylt	female	n.a.	severe	4.00	flatfish	–
25	95∕07∕07	North Sea, Sylt	female	n.a.	moderate	7.50	Common sole	pharynx
26	98∕04∕15	North Sea, Sylt	female^*^	good	good	10.00	flatfish	–
27	98∕04∕28	North Sea, Sylt	female^*^	moderate	good	12.00	flatfish	–
28	00/–/–	North Sea, Pellworm	male	good	moderate	7.00	flatfish	pharynx/nasal passage
29	02∕01∕01	North Sea, North Friesland	female	good	good	adult	flatfish	–
30	07∕04∕12	North Sea, Sylt	female	good	fresh	adult	Common sole	–
31	08∕04∕14	North Sea, Sylt	male	good	good	6.00	flatfish	–
32	09∕05∕22	North Sea, Sylt	female^*^	moderate	moderate	16.00	flatfish	–
33	10∕05∕16	North Sea, Sylt	male	good	good	8.00	Common sole	pharynx
34	15∕05∕10	North Sea, Sylt	male	moderate	moderate	adult	flatfish	pharynx
35	16∕08∕29	Baltic Sea, Laboe	female	–	severe	adult	Codfish	pharynx
36	16/–/–	–	female	good	good	adult	flatfish	pharynx
37	17∕04∕15	North Sea, Sylt	female^*^	moderate	fresh	6–7	flatfish	pharynx/nasal passage
38	17∕04∕26	Baltic Sea, Booknis	female	moderate	moderate	adult	flatfish	pharynx
39	17∕06∕29	North Sea, Eider barrier	male	–	severe	juvenil	flatfish	pharynx/nasal passage

**Notes.**

* pregnant.

** lactating.

n.a. not assessed.

- no specification.

**Table 5:** Percentage of fatal pharyngeal entrapment of fish. Total number of investigated harbour porpoises from the coasts of Schleswig-Holstein from 1990 to 2019 as well as the number of all carcasses coming from the North Sea coast, the number of animals having fatal pharyngeal entrapment of fish and of these, the total percentage of animals derived from the North Sea coast.

**Stranding year**	**Total number**	**North Sea**	**CasesNorth Sea**	**North Sea (%)**
1990	16	6	2	33.3
1991	43	21	6	28.6
1992	26	19	5	26.3
1993	34	27	3	11.1
1994	38	28	3	10.7
1995	32	21	6	28.6
1996	28	15	0	0
1997	22	17	0	0
1998	48	40	2	5
1999	17	13	0	0
2000	20	16	1	6.3
2001	29	23	0	0
2002	22	14	1	7.1
2003	27	25	0	0
2004	38	28	0	0
2005	31	27	0	0
2006	46	39	0	0
2007	37	21	1	4.8
2008	48	19	1	5.3
2009	32	19	1	5.3
2010	21	9	1	11.1
2011	28	8	0	0
2012	30	22	0	0
2013	29	15	0	0
2014	27	17	0	0
2015	49	23	1	4.3
2016	86	37	9	24.3
2017	193	95	2	2.1
2018	206	72	0	0
2019	230	84	0	0
**in total**	**1,533**	**820**	**45**	**6.3**

**Table 6:** Monthly occurrence of cases. Number of cases of fatal pharyngeal entrapment of fish in harbour porpoises from the German North Sea from 1990–2019 listed per month and the percentage of the total number of cases with known stranding date ( *n* = 41).

	**Number of cases**	**Percentage per month (%)**
**January**	1	2,4
**February**	0	0
**March**	0	0
**April**	9	21,9
**May**	14	34,1
**June**	6	14,6
**July**	5	12,2
**August**	3	7,3
**September**	3	7,3
**October**	0	0
**November**	0	0
**December**	0	0

**Figure 6:**
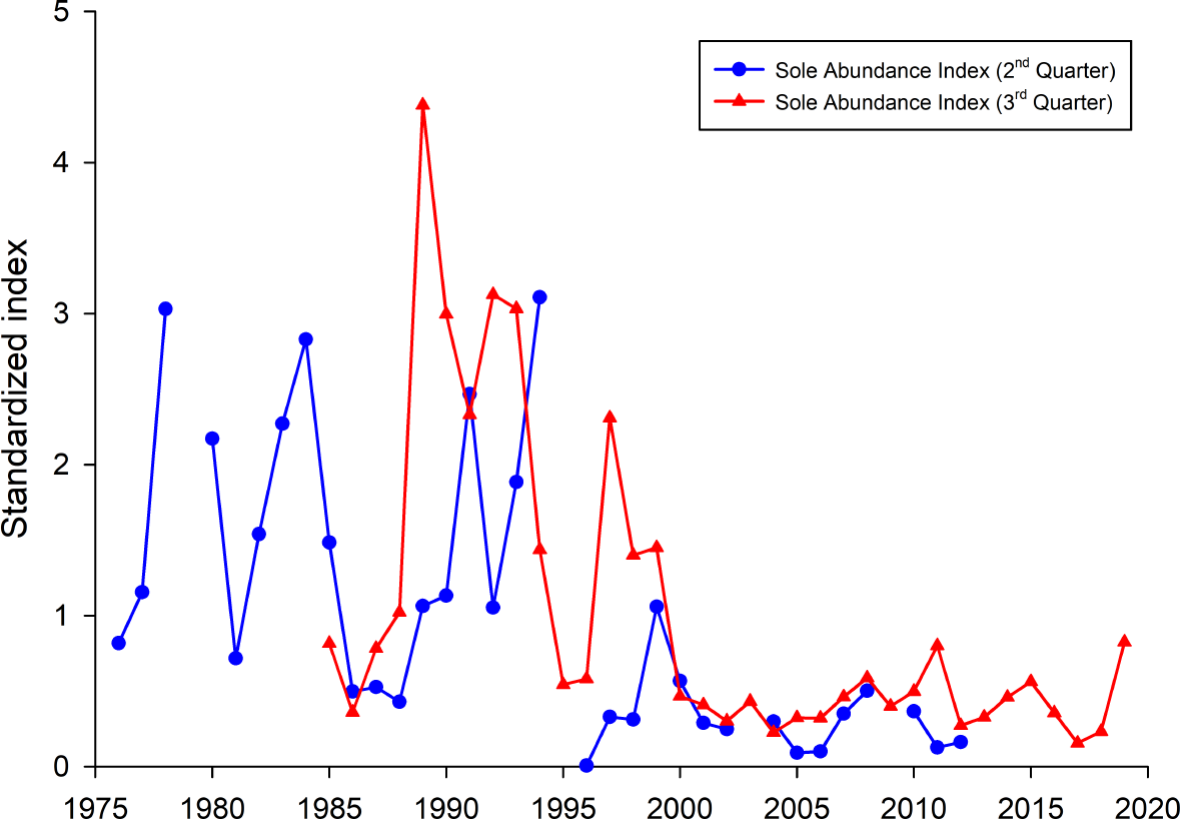
Sole abundance indices (standardized by the mean) for the German Bight area. The 2nd quarter index (blue line) is based on the German sole survey (1976 –2012). The 3rd quarter survey is based on data from the North Sea beam trawl survey (BTS).

To investigate if the occurrence of common sole was, compared to all other years, higher in the years 1990–1992, 1995 and 2016, fish survey data were analysed (Fig. 6). Both presented sole indices for the German Bight area are highly variable and show contradicting trends for some years, especially in the earlier part of the time series. It has to be noted here that due to the different survey designs and seasonal effects, the two indices cannot necessarily be compared directly. Since all age classes are included in these indices one source of the observed variability is incoming recruitment. However, both indices display the general trend with higher observed abundances before the year 2000 and relatively low values since then.

## Discussion

The present study describes an unusual spatiotemporal accumulation of fatal pharyngeal entrapment of common sole in nine harbour porpoises that were found stranded on the German North Sea island of Sylt in 2016. Post-mortem examination revealed a dislocated larynx associated with the flatfish obstructing the pharynx. With regard to choking there are two lethal mechanisms described in the literature. The first one is asphyxiation due to a mechanical obstruction of the airway by food items or foreign bodies which inhibit respiration (McEwen, 2016). A different lethal mechanism described in humans is the cardiac arrest due to reflex vagal inhibition which is also known as bolus death and can be caused by food items stimulating the superior laryngeal nerve (Althoff & Dotzauer, 1976). It was not possible to differentiate if the fatal outcome in the harbour porpoises was caused by suffocation or cardiac arrest or a combination of both mechanisms. None of the cases displayed evidence of any other cause of death. In all nine cases, a common sole obstructed the airways. In seven cases, the fish partly stuck in the internal nares. In one case, the fish had gone down the trachea and in another one the fish was bent between the caudal part of the oral cavity and the larynx. In addition, a retrospective analysis of data from the ITAW revealed a similar case accumulation on the same island and the close surroundings in the years 1990, 1991, 1992, and 1995. In this data analysis, harbour porpoises that had stranded dead on the North and Baltic Sea coasts of the German federal state Schleswig-Holstein were included. These animals belonged to two different populations, namely the North Sea population and the Western Baltic Sea population (Wiemann et al., 2010; Galatius, Kinze & Teilmann, 2012; Lah et al., 2016).

All of the reported airway obstructions here that occurred on the German North Sea coast were caused by flatfish. Of these 45 cases, 36 fish (80%) were identified as common sole, whereas the remaining nine were unidentified. This clearly indicates common sole as a risky item of prey for harbour porpoises in this area. The risk seems not only to be dependent on the size of the fish. Two previous studies identified the usual size of ingested sole in harbour porpoise from the North Sea as varying between 13.2 to 17.6 cm (Gilles, 2008) and 10 to 20 cm (Benke et al., 1998), respectively. Thus, with lengths varying between 16.7 and 28.8 cm, some of the soles in this study were within the size range of successfully ingested sole. Furthermore, common sole has a slender body shape compared to other flatfish species (Muus & Nielsen, 2013) which should allow them to pass the pharyngeal bottleneck more easily. Thus, other factors, like their high agility and flexibility (Kruuk, 1963; IJsseldijk et al., 2015) must also be taken into account for the high tendency of soles to cause fatal incidences in harbour porpoises. These skills help the sole in their attempt to escape being ingested but may cause obstruction when they find a potential escape route or flap around in the upper digestive tract. In the latter case, the lateral spines on the fish body increase the risk of the fish being trapped in the upper respiratory or digestive tract of its predator. In none of the cases described above were macroscopical changes like trauma seen in the oesophageal mucosa. Thus, no indication was evident that the spines of the fish were involved in the obstruction. The fish may also survive longer than the porpoise while trying to find a way out. Therefore, it is possible that the location where the fish was found during post-mortem examination might not be the same as that that caused the fatal pharyngeal obstruction.

Especially in humans, it is described that the act of swallowing can be impaired by neurological alteration, being inebriated and drug intake, all of which can thereby lead to fatal asphyxiation or bolus-death (Althoff & Dotzauer, 1976; Bockholdt, Ehrlich & Maxeiner, 2003; Wick, Gilbert & Byard, 2006). As heavy metals and other chemicals have been shown to cause neurological alterations (Jacobs & LeQuesne, 1984), toxicological screening could have provided additional information. This should include marine biotoxins like domoic acid which also have been shown to cause neurological signs in cetaceans (De la Riva et al., 2009). Due to the decomposition status, only in eight cases a histopathological investigation of the brain has been performed. In these cases no evidence for a neurological disease was found. Anyway, it seems to be unlikely that the cases presented here were associated with neurological impairment due to disease or intoxication as otherwise a variation in involved fish species would be expected. This highlights again the special role common sole may have as a potential fatal item of prey for harbour porpoises.

Another interesting point, besides the fact that here only common sole was identified as a causative agent, is the high seasonality of the cases in the North Sea. More than 80% occurred from April to July with a peak in May with 34.1% of all cases (14/41). It is noteworthy that the majority of harbour porpoise strandings occur on the coasts of Schleswig-Holstein between June and August (Hasselmeier et al., 2004; Siebert et al., 2006), with strandings in winter months being very rare. If the occurrence of fatal pharyngeal entrapment correlated with the number of stranded carcasses, most findings would have occurred in July and August. Potential explanations may be an increased abundance of common sole or a decrease in other prey species during spring and early summer. Interestingly, one study investigating the feeding ecology of harbour porpoises in German waters between 1994 and 2006 found a significant seasonal variation in prey species (Gilles, 2008). Hard-parts analysis from stomach contents indicated that harbour porpoises consume common sole mainly in winter and spring, less in summer and not during autumn (Gilles, 2008). This correlates with the present study in regard to seasonal accumulation of fatal pharyngeal obstruction caused by common sole during springtime. Besides seasonal variation, interannual fluctuations in common sole consumption were also noted. Common sole was mainly identified in the years from 1994 to 2000. After that, only few common soles were found and only in 2004 and 2006 (Gilles, 2008). On the whole, this correlates with fishery data which show high landings of common sole for the German North Sea in the years 1990 to 1995 (ICES, 2019). In 1999 and 2000, landing numbers were relatively high again but afterwards decreased significantly. The spawning-stock biomass of common sole was also highest in the first half of the 1990s (ICES, 2019). This is in line with the here presented sole indices. Thus, the high number of observed fatal obstructions in the first half of the 1990’s can partly be explained by relatively high observed sole abundances in the German Bight during that time period. However, this is not the case for the year 2016 as since 2000 the sole abundances were on a comparable low level. This indicates that other mechanisms, e.g., lack of preferred prey species, may be responsible for the observed high incidences of fatal pharyngeal entrapment of fish in harbour porpoise. This may account for the increase in the consumption of sole and therefore the risk of them obstructing the airways in harbour porpoises. In general, common sole seem to be ingested successfully as they are regularly found in the stomach of harbour porpoises (Benke et al., 1998; Gilles, 2008). Fatal pharyngeal entrapment may occur in unfortunate circumstances where the common sole is either too large or too agile. The low occurrence of cases in the Western Baltic Sea harbour porpoise population might be attributed to the lack of common sole in most parts of the Baltic Sea, occurring only in the waters of the Skagerrak and Kattegat (Muus & Nielsen, 2013). The two Baltic Sea cases were caused by an unidentified flatfish and a codfish, respectively.

A survey of the seasonal distribution of harbour porpoises in the German North Sea highlights the Sylt Outer Reef (SOR) as the most important focal region (Gilles, Scheidat & Siebert, 2009). Harbour porpoises aggregate during spring mainly in two areas, the SOR and the Borkum Reef Ground, while during summer, the SOR is the only aggregation hot spot. In autumn, the animals spread and are more evenly distributed. In general, harbour porpoise numbers increase during spring, reach a peak in May and June, before decreasing in autumn and occurring only in low numbers during winter. Aggregation hot spots were assumed as areas of high prey availability (Gilles, Scheidat & Siebert, 2009). This corresponds well with the general stranding frequency, but most fish-related deaths occurred during spring and early summer, pointing again to a potential increase in common sole abundance.

So far, only one other study reported on the occurrence of fatal pharyngeal entrapment of fish in separate odontocete populations (Stolen et al., 2013). Here, necropsy data on bottlenose dolphins inhabiting the Indian River Lagoon in Florida, USA, were reviewed over a 15-year period from 1997 to 2011. With 4% fish-related deaths (14/350) in one population, the overall incidence was lower than in the present study (5.5%). In Florida, USA, three different fish species were identified to have caused fatal pharyngeal entrapment, two of them having strong dorsal spines. Furthermore, in five of the cases, recreational fishing gear was also involved in the obstruction. No seasonality or fluctuation between the years is reported. Additionally, 186 cases of an adjacent dolphin population were reviewed, showing no case of fish-related death. The different occurrence was assumed to be related to differences in prey abundance and distribution and/or differences in the foraging ecology of the two dolphin populations. Different prey species are also assumed to be responsible for the different occurrence of fatal cases in the two harbour porpoise populations in German waters. Due to the seasonality and annual fluctuation of cases, differences in foraging behaviour seem to be an unlikely reason for the described cases in Germany. Although, to our knowledge, no detailed data on seasonal and annual abundance of different prey fish species of harbour porpoises exist for German waters, intra- and inter-yearly variations in the abundance of different fish species are most likely responsible for the reported temporal accumulation of fatal pharyngeal entrapment caused by common sole in the North Sea harbour porpoise population.

## Conclusion

The phenomenon of fatal pharyngeal entrapment of fish in odontocetes is well known, however mainly single cases have been reported from different parts of the world involving various fish species. This study evaluates the occurrence of fatal pharyngeal entrapment of fish in two harbour porpoise populations from the coasts of Schleswig-Holstein over a 30-year period. This phenomenon was identified as the cause of death in 0.3% of all examined harbour porpoises from the Western Baltic Sea population and in 5.5% of the animals belonging to the North Sea population. In the North Sea population, a spatiotemporal accumulation of these incidents occurred. Most cases were found stranded during spring and early summer on the island of Sylt and in surrounding regions. Although single cases intermittently occurred over the whole time span, especially the years 1990–1992, 1995 and 2016 stand out with an occurrence of 24.3 to 33.3%. All flatfish where the species could be identified were common sole, indicating this species as a potentially risky item of prey for harbour porpoises. The low occurrence of fatal pharyngeal entrapment of fish in the Western Baltic Sea population is attributed to the absence of common sole in this area. The seasonal and annual variation in cases in the North Sea might be caused by an increased intake of common sole either due to a higher abundance of this fish or a decrease in other prey species.

##  Supplemental Information

10.7717/peerj.10160/supp-1 Supplemental Information 1 Raw data for Fig. 1 The numbers per hour trawling (“n_hour”) per fished station with latitutde and longitude and the respective ICES statistical rectangle (“StatRec”) as displayed in Fig. 1. Further, the ship code, country, quarter of the year, station and haul number is listed. Only data from the ICES statistical rectangles 39F7, 39F8, 38F7, 38F8, 37F7, 37F8 were used. The per haul raw data of the BTS (Beam Trawl Survey) are also available via the ICES DATRAS data portal and can be downloaded in the DATRAS exchange format.
Year, Year of sampling; Ship, DATRAS ship reference code; StatRec, ICES statistical area rectangle (0.5 degrees latitude, 1 degree longitude); Shooting-position sensitive; StNo, Station number; National coding system; not defined by ICES; HaulNo, Sequential numbering of hauls during cruise; Quarter, Cruise quarter of the year; Country, DATRAS code for the country that performed the survey; ShootLat, Hauling position: Degree.Decimal Degree of latitude; ShootLong, Shooting position: Degree.Decimal Degree of longitude; n_hour, Number of sole per hour trawling; Station_ID, Unique station ID

10.7717/peerj.10160/supp-2 Supplemental Information 2 Raw data for Fig. 6 The calculated relative index values of the German sole survey and the BTS (Beam Trawl Survey). This index is calculated as the mean number of sole per hour trawling for each year in the investigation area. The index values for each time series were standardized by the mean and displayed in Fig. 6.

## Additional Information and Declarations

### Data Availability

The following information was supplied regarding data availability: Data is available at ICES (2020a): http://dx.doi.org/10.17895/ices.pub.6028. Measurements are available in the Supplemental Files.
